# A Novel Technique of Contrast-Enhanced Optical Coherence Tomography Imaging in Evaluation of Clearance of Lipids in Human Tears

**DOI:** 10.1371/journal.pone.0109843

**Published:** 2014-11-04

**Authors:** Pietro Emanuele Napoli, Franco Coronella, Giovanni Maria Satta, Maurizio Fossarello

**Affiliations:** Department of Surgical Sciences, Eye Clinic, University of Cagliari, Cagliari, Italy; University of Zurich, Switzerland

## Abstract

**Purpose:**

The aim of this work was to gather preliminary data in different conditions of healthy eyes, aqueous tear deficient dry eyes, obstructive meibomian gland disease (MGD) and non-obvious obstructive MGD (NOMGD) individuals, using a new, contrast-enhanced optical coherence tomography (OCT) imaging method to evaluate the clearance of lipids in human tears.

**Methods:**

Eighty-two adult patients presenting with complaints of ocular irritation were studied for abnormalities of the ocular surface and classified as healthy (n = 21), aqueous tear deficient dry eyes (n = 20), obstructive MGD (n = 15) and NOMGD (n = 26) individuals. A lipid-based tracer, containing an oil-in-water emulsion, was used to obtain an enhanced OCT imaging of the lower tear meniscus. After instillation, a dramatic initial increase of reflectivity of the lower tear meniscus was detected by OCT, followed by a decay back to baseline values over time. Based on this finding, the clearance of lipids was measured in real-time by Fourier-domain anterior segment OCT.

**Results:**

The differences in the clearance of lipids among the four groups as well as the correlations between symptom questionnaire score, standardized visual scale test, fluorescein break-up time, ocular surface fluorescein staining score, Schirmer I test scores were found to be statistically significant. The individual areas under the curve of the clearance of lipids calculated by the receiver operating characteristic curve technique ranged from 0.66 to 0.98, suggesting reliable sensitivity and specificity of lipid-enhanced OCT imaging.

**Conclusions:**

This new technique of contrast-enhanced OCT imaging of the tear film following lipid-based tracer instillation provides a measure of the clearance of lipids. The quantitative values found are in agreement with other methods of evaluation of the lacrimal system. An improvement of the clinician's ability in the diagnosis and understanding of abnormalities of the ocular surface may be achieved by this simple approach.

## Introduction

The current model of the precorneal tear film consists of a thick aqueous-mucin layer covered by a thin lipid layer. The lipid layer is the most anterior layer of the tear film, composed of meibomian lipids that limit tear evaporation and stabilize the tear film. [1] In case of absence or altered integrity of the lipid layer, the evaporation rate of tears increases, and produces tear-film instability. [2], [3] In fact, the lipid layer measured by interferometry has been reported to correlate with tear-film evaporation, tear-film break-up time, and clinical symptoms. [4], [5]

In various pathological conditions, such as meibomian gland dysfunction (MGD), the appearance of the lipid layer may change. A meibomian lipid deficiency due to obstructive MGD is an area of growing clinical interest since it is now recognized to be the most common cause of evaporative dry eye. [6], [7] The latest clinical classification of the ocular surface disorders includes a type of obstructive MGD that is obvious upon examination, and a non-obvious obstructive meibomian gland dysfunction (NOMGD). [8] NOMGD is potentially considered the most common form of obstructive MGD, but it is frequently missed during clinical examination, since it starts with minimal signs and symptoms, requiring clinical evaluation of meibomian gland expressibility for its diagnosis.

Although several studies have assessed the flow of the aqueous layer by means of the clearance of fluorescein sodium, the turnover or clearance of lipids (CoL) in the tears of patients with abnormalities of the ocular surface has not been yet documented in literature. [9]–[18] Since fluorescein sodium is highly water-soluble at physiologic pH, its clearance is essentially an index of the aqueous tear turnover. However, the elimination rate of other tear components does not parallel the aqueous tear clearance. [19]

Recently, optical coherence tomography (OCT) has been used to obtain detailed cross-sectional images of anterior tissues of the eye and to assess the tear film. [20]–[24] OCT imaging may potentially permit observation of fine details of the ocular surface and understanding new aspects about the behavior of human tears in vivo.

In the present study, we used Fourier-domain anterior segment OCT to analyze the dynamic distribution of lipids in the lower tear meniscus and to determine the CoL in humans. For this purpose, we administered a lipid-based tracer to enhance the visibility of lipids and to track their flow in the lower tear meniscus with OCT. In this way, we evaluated the correlation between the CoL and aqueous tear turnover as well as classical tear tests, and the diagnostic validity of this new technique of contrast-enhanced OCT imaging, in healthy subjects and in patients with aqueous tear deficiency (ATD), obstructive MGD and NOMGD.

## Materials and Methods

### 1. Subjects and procedure

Adult patients presenting with complaints of ocular irritation were evaluated at the Eye Clinic, Department of Surgical Sciences and Odontostomatology, University of Cagliari, School of Ophthalmology. Written informed consent for participation was obtained from all subjects, after ethics approval obtained from the Office of Research Ethics, University of Cagliari. The study complied with the guidelines in the Declaration of Helsinki for research involving human subjects.

All examinations were conducted in the same conditions of temperature (within a range of 15°C to 25°C), humidity (within a range of 30% to 50%) and time of the day (between 3 PM to 5 PM) in a dimly lit consulting room.

All subjects completed a symptom questionnaire (OSDI  =  Ocular Surface Disease Index) [25] consisting of a set of questions assessing the level of discomfort and the functional impact of their irritation symptoms.

On the day before OCT imaging, a standard clinical assessment was performed on all subjects in the same sequence. It included: clinical history, fluorescein break-up time (FBUT), fluorescein staining of the cornea and conjunctiva graded according to the Oxford system, [26] standardized visual scale test (SVST), Schirmer I test, and a slit lamp examination of the lid margins and meibomian glands.

Since it is good practice to work from the least invasive to the most invasive test, [26] OCT imaging was carried out a day after the clinical assessment, in order to avoid one tear test interfering with the CoL, as well as to prevent manipulation disrupting the tear film/ocular surface.

Based on the results of these tests, patients were classified into one of four groups based on the following inclusion criteria:

#### 1.1. Aqueous tear deficiency

If the subject exhibited all of the following characteristics: significant subjective symptoms (OSDI score ≥13), a FBUT <10 seconds, a significant vital staining of the ocular surface (Oxford scheme ≥2 or panel B) and a Schirmer I test ≤5 mm.

#### 1.2. Obvious Obstructive meibomian gland disease

It was considered to be present when the patient exhibited Schirmer I test>5 mm and all of the following three signs/findings: [27]

- Chronic ocular discomfort (OSDI score ≥13).
- Anatomic abnormalities around the meibomian gland orifices (presence of one or more of the following is positive):
  - irregularity of the lid margin;
  - vascular engorgement;
  - anterior or posterior displacement of the mucocutaneous junction.
- Obstruction of the meibomian glands (presence of both is considered positive):
  - decreased meibum expression by moderate digital pressure;
  - obstructive findings of the gland orifices by slit lamp biomicroscopy (pouting, plugging, or ridge).

#### 1.3. Non-obvious obstructive meibomian gland disease

It was diagnosed only after gland expression [8] (gland orifices failing to yield any secretion on expression), in patients with asymptomatic dysfunction or occasional symptoms (OSDI <12), Schirmer I test>10 mm and normal lid margin features.

#### 1.4. Healthy

Subjects with no history of use of eyedrops, no significant symptoms of ocular irritation (OSDI <12), FBUT>10 seconds, Oxford scheme ≤ panel A, and a Schirmer I test score more than 10 mm were considered as healthy.

Subjects with other *abnormalities* of the ocular surface or of the tear film, other eye diseases in the previous 6 months, excessive meibomian lipid secretion (meibomian seborrhea) or any evidence of abnormal blinking, history of contact lenses wear or of eye surgery, were excluded from the study.

### 2. FBUT and fluorescein ocular surface staining

In order to enhance the observation of dry spots in the fluorescent tear film, over the entire cornea, and the conjunctival staining, the ocular surface was examined with a biomicroscope and the ×10 objective under both blue-light illumination and Kodak Wratten 12 yellow filter, within 10–30 seconds (for FBUT) and after 2 minutes (for grading staining) of fluorescein sodium instillation (t = 0). Three evaluations of FBUT were conducted, and the mean value was taken for data analysis. Staining of the ocular surface was graded according to the Oxford system as follows: 1 =  panel A, 2 =  panel B, 3 =  panel C, 4 =  panel D, 5 =  panel E, 6 =  panel>E.

### 3. Standardized Visual Scale Test (tear fluorescein clearance)

The SVST was performed as previously reported. [28] Briefly, the color of the tear meniscus in the third lateral of the lower lid was visually compared with one of the colors of the standardized visual scale. If the color of the tear meniscus was judged to be between two of the six standard scale colors, then the score was graded between these two standard colors.

After the SVST, Schirmer I test and the slit lamp examination of the lid margins and meibomian glands were performed.

### 4. Lipid-based tracer

The castor oil is a vegetable oil containing a mixture of lipids (approximately 90 percent of fatty acid chains are ricinoleate), which mimics the oily lipid secretions produced by meibomian glands, i.e. the natural lipids in the tear film. In fact, for this reason it is contained in some lipid-based artificial tears. The lipid-based tracer was obtained in the following way: a castor oil emulsion (0.50%) was prepared in sterile 0.067 M phosphate-buffered saline, pH 7.4, with Tween 80.

**Figure 1:**
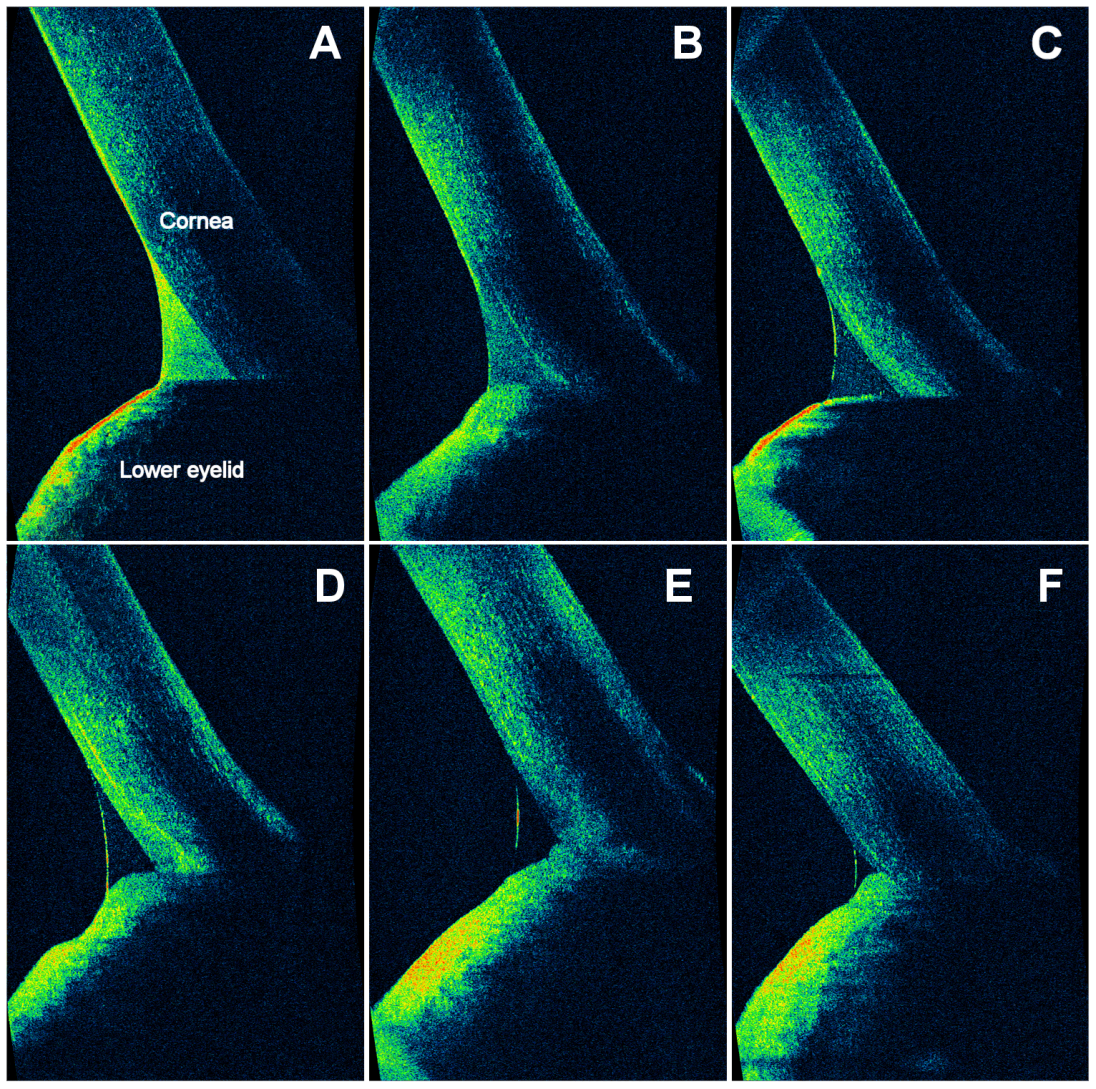
Association between OCT reflectivity and amount of lipids. Vertical, 3-mm OCT scans of the lower tear meniscus. The lipid-based tracer containing different concentrations of castor oil (0.5%, 0.25%, 0.125%, and 0.06%) or saline solution were instilled in five subjects (A, B, C, D, and E, respectively). OCT imaging revealed a positive correlation between the signal intensity detected by OCT and lipids concentration. At baseline (F), the reflectivity in the lower tear meniscus was nearly undetectable.

**Figure 2:**
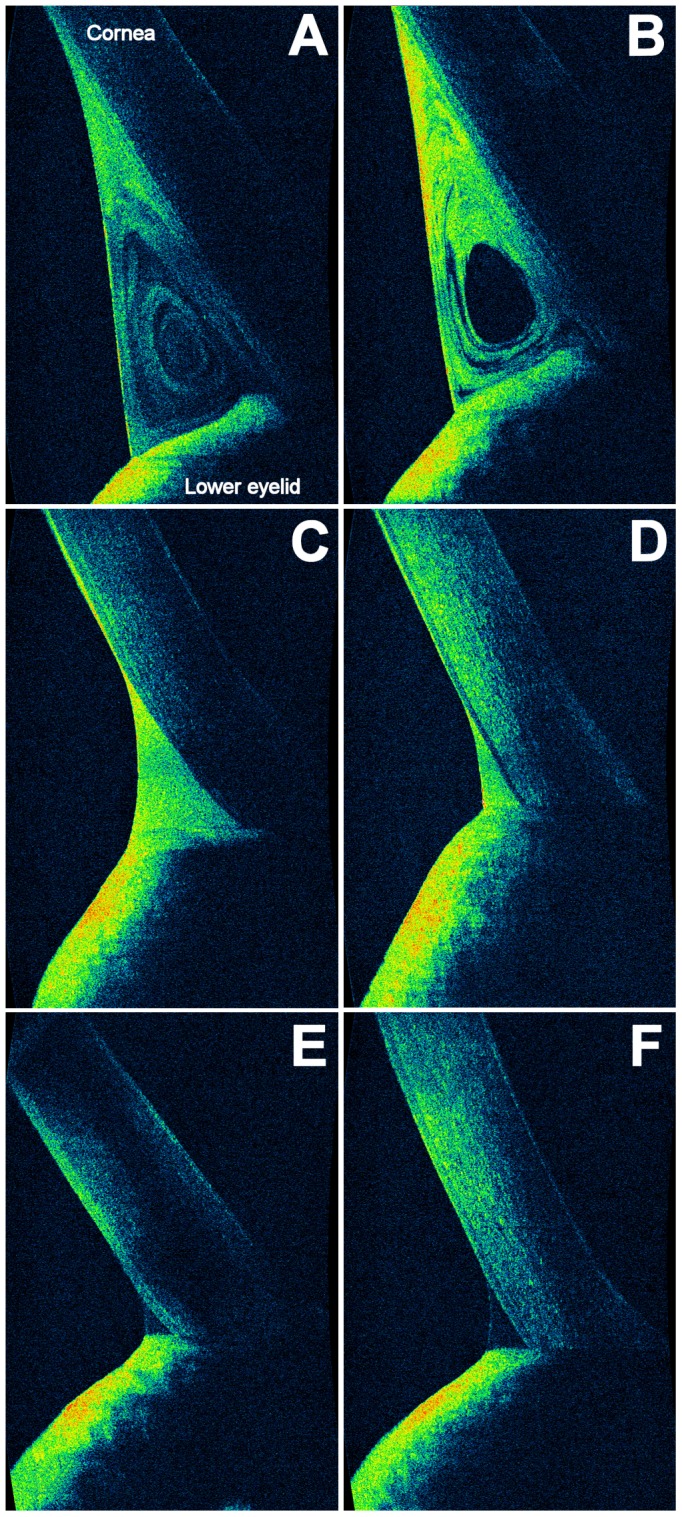
OCT images of the different patterns of the lower tear meniscus. Vertical, 3-mm OCT scans. The cross-sectional ocular surface images were acquired during normal blinks and evaluated in real-time. A dramatic initial increase of reflectivity in the lower tear meniscus was detected by OCT after instillation of the lipid-based tracer, followed by a decay back to baseline values over time. The reflectivity of the lower tear meniscus at the 15^th^ minute was considered an index of turnover of tear lipids. The tracer was distributed in the lower tear meniscus according to the two morphological patterns: a vortex type (A and B), which shows areas of high/moderate reflectivity distributed concentrically around a central area of low/absent reflectivity, and a homogeneous type (C, D, E and F), which shows areas of similar reflectivity in the whole lower tear meniscus, classified according to the reflectivity grading scale. At instillation (C and D), the reflectivity was high/intense in all patients. At baseline (E and F), the reflectivity detected by OCT in the lower tear meniscus was nearly undetectable.

A preliminary experiment was performed to test the association between OCT reflectivity and the amount of lipids. Immediately after instillation of 35 µl of tracer containing various concentrations of castor oil (0.5%, 0.25%, 0.125%, 0.06%) or saline solution (35 µl, Blu Sal, Sooft, Italy) into five subjects' eyes was performed OCT imaging. Thus, a positive correlation between OCT reflectivity and lipids concentration was revealed (fig 1). Castor oil concentration of 0.50% offered the greatest clarity of details and, therefore, was used as tracer. Accordingly, the tracer (castor oil 0.5%) was detected by OCT as a dramatic initial increase of reflectivity in the lower tear meniscus, followed by a decay back to baseline values over time (fig 2). In this way, we obtained a lipid-enhanced OCT imaging. No signs of inflammation or damage were detected either immediately or after 24h.

**Figure 3:**
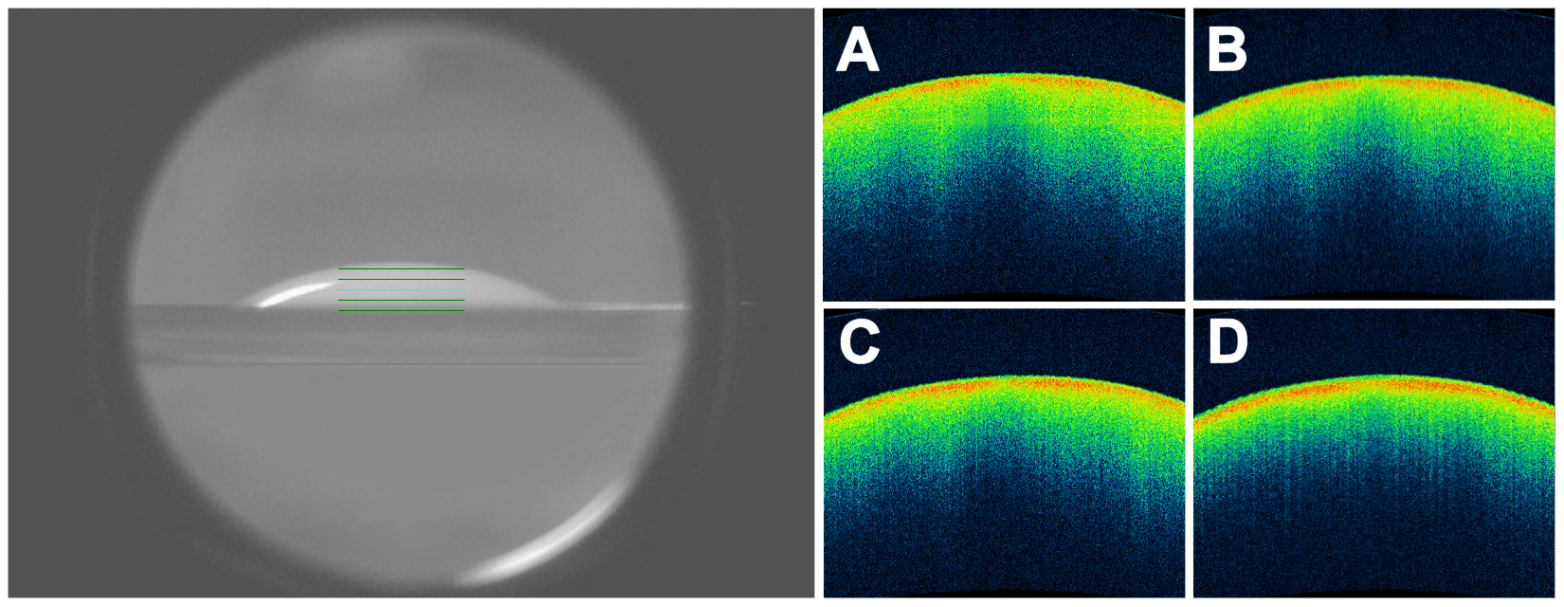
OCT serial images of a calibrated drop of the lipid-based tracer (35 µl, castor oil 0.5%) on a glass slide. Infrared image (left), and horizontal, 3-mm OCT scans (right). The OCT scans were performed at instillation, at the 5^th^, at the 10^th^ and at the 15^th^ minute (A, B, C and D, respectively). No changes in reflectivity of the tracer were observed in this *control* sequence of contrasted-enhanced OCT imaging.

To test the stability of the tracer in the absence of tear clearance (in order to obtain a *control* sequence of contrast-enhanced OCT imaging), a drop of the lipid emulsion (35 µl, castor oil 0.5%) was placed on a glass slide and its reflectivity was evaluated at instillation, at 5 minutes, at 10 minutes and at 15 minutes (i.e., at the same time-points of OCT examination, as described below). As expected, the tracer remained hyper-reflective for all the time of the test (fig. 3).

### 5. OCT measurements and scoring

The lower tear meniscus of one randomly chosen eye in each patient was imaged by vertical scans on the vertical axis passing across the corneal apex. OCT scans were performed by using Cirrus™ HD-OCT 4000 (Carl Zeiss Meditec Inc., Dublin, California, USA). This system is a Fourier-domain OCT platform that works at a wavelength of 840 nm, takes 27,000 axial scans per second and has a 5 µm axial resolution. The cross-sectional ocular surface images were acquired using the Anterior Segment 5 Line Raster scanning protocol. The mode acquires a set of five parallel lines of equal length at 3 mm. Each scan line comprised 4096 A-scans with the overall 5 Line Raster scan taking approximately 0.75 seconds to complete. For our purposes, the scan lines were orientated vertically and each separated horizontally by 250 µm. After image capture, the individual line with greatest clarity of detail was selected for our analysis. The subject was asked to blink normally during the examination period. Before each scan, patients were instructed to look straight ahead. The lower tear meniscus was imaged 2 seconds after a blink. The axial distance of patients was adjusted so that the lower tear meniscus was within the middle third of the scan. [20]

The evaluation of the CoL in human tears was performed by analyzing the clearance of the lipid-based tracer (35 µl) in the lower tear meniscus.

The OCT scans were performed at baseline and after the instillation of the tracer in four serial scans: immediately (within 30 seconds), at the 5^th^, at the 10^th^ and at the 15^th^ minute. Thus, we have classified the tear clearance of lipids, with respect to the apparent reflectivity, according to the following grading scale: grade 1 or excellent (black  =  absence of reflectivity), grade 2 or good (from black to blue  =  low reflectivity), grade 3 or fair (from blue to green  =  moderate reflectivity), grade 4 or poor (from green to yellow/red  =  high/intense reflectivity).

The reflectivity of the lower tear meniscus at the 15^th^ minute was considered an index of turnover of tear lipids.

The dynamic distribution of the tracer was also classified according to the morphological pattern in two different categories (fig. 2): a homogeneous type (areas of similar reflectivity in the whole lower tear meniscus, classified according the previous grading scale) and a vortex type (areas of high/moderate reflectivity distributed concentrically around a central area of low/absent reflectivity).

All information were recorded anonymously, so that subjects could not be identified, directly or through identifiers linked to the subjects.

### 6. Index of Lipid/Aqueous dysfunction

To improve the sensitivity of our test in differentiating the impact of a lipid alteration from an aqueous deficiency, the following tear dysfunction index (TDI) was introduced: TDI  =  CoL grade/Schirmer I test grade

The lipid turnover at the 15^th^ minute was graded as follows: 1 =  excellent, 2 =  good, 3 =  fair, 4 =  poor. Since the Schirmer test score is also a discrete variable, [6] as wetting length values are taken as the nearest integer or half integer rather than as continuous fractions of a millimeter, it was evaluated, considering the severity of the aqueous deficiency, [6] in four ranks: 1 =  normal (score >10 mm), 2 =  borderline (score between 6 mm and 10 mm), 3 =  abnormal (score between 5 mm and 3 mm), 4 =  abnormal with considerable severity (score ≤ 2 mm).

The TDI has a precise rationale: to compare the influence of a lipid alteration with respect to an aqueous deficiency. Therefore, the TDI does not aim to differentiate a normal subject from a dry eye patient, which is the role assigned to tear tests. The best cutoff point (y) for the TDI was calculated by analyzing the receiver operating characteristic curves (ROCs). Thus, the TDI score was considered as follows: predominant lipid alteration (TDI score> y), predominant aqueous deficiency (TDI score < y).

### 7. Statistical analysis

Statistical analysis was performed using Statistical Package for Social Science SPSS version 21.0. Numeric data were summarized as mean ± standard deviation (SD) for parametric data, as median and mode ± average absolute deviation for ordinal data, while nominal data were summarized as percentage.

Data were analyzed by Shapiro-Wilk test and Lilliefors test for normality. If data were normally distributed, then parametric statistical tests were used, otherwise nonparametric tests were used. Particularly, the difference in age among the four groups was studied by means of one-way analysis of variance, and Bonferroni correction for the *post-hoc* analysis. The difference in gender, OSDI, FBUT, fluorescein staining of the ocular surface, SVST, Schirmer I test, CoL and TDI, among the four groups was studied using the Kruskal-Wallis test, followed by *post-hoc* Mann–Whitney U tests with Bonferroni correction to identify specific differences between each group.

Diagnostic results for dry eye were analyzed by receiver operating characteristics (ROC) curve, and the area under the ROC curve (AUC) was calculated. Other specific statistical tests are described as they are encountered in the article. P-values less than 0.05 were considered significant.

## Results

### 1. Differences in CoL among the four groups

No significant differences in age and gender were found between control participants (n = 21; age: 45.89±15.14 years, 90.5% female), patients with ATD (n = 20; age: 45.10±13.95 years, 90% female), patients with obstructive MGD (n = 15; age: 49.13±19.50 years, 86.7% female), and patients with NOMGD (n = 26; age: 46.88±16.82 years, 88.5% female). Descriptive statistics for diagnostic tests, as well as the statistically significant differences among the four studied groups are provided in Table 1 and 2.

**Table 1:** Statistical Comparison of four Groups: Healthy, Aqueous Tear Deficiency (ATD), obvious obstructive Meibomian Gland Dysfunction (MGD), and Non-obvious Obstructive MGD (NOMGD) Patients.

Group	OSDI†	FBUT*	FSOS grade†	SVST grade†	Schirmer 1 test*	CoL grade (15^th^ minute)†	TDI*
							
Healthy persons	4.2±3.3	10.7±4.2	1±0.0	1±0.1	23.6±10.7	1±0.9	1.3±0.4
(n = 21)							
ATD patients	47.4±16.7	5.1±2.9	3.(4)±0.1	6±0.8	1.9±1.5	4±0.5	0.9±0.1
(n = 20)							
Obstructive MGD patients	25.2±3.1	5.1±1.3	2±0.0	4±0.6	11.9±7.3	4±0.0	2.8±1.0
(n = 15)							
NOMGD patients	6.5±4.1	8±1.1	1±0.0	4±0.1	17.7±11.4	3±0.0	3.0±0.0
(n = 26)							
Differences among	K = 65.3	K = 45.7	K = 51.0	K = 61.2	K = 52.9	K = 65.8	K = 57.3
the four groups	*p*<0.001	*p*<0.001	*p*<0.001	*p*<0.001	*p*<0.001	*p*<0.001	*p*<0.001
Differences between	U = 0.0	U = 37.0	U = 63.0	U = 0.0	U = 0.0	U = 0.0	U = 123.5
healthy and ATD	*p*<0.001	*p*<0.001	*p*<0.001	*p*<0.001	*p*<0.001	*p*<0.001	*p* = 0.009
Differences<between	U = 0.0	U = 0.0	U = 21.0	U = 16.0	U = 60.0	U = 0.0	U = 36.0
healthy<and<MGD	*p*<0.001	*p*<0.001	*p*<0.001	*p*<0.001	*p* = 0.002	*p*<0.001	*p*<0.001
Differences<between	U = 181.0	U = 188.0	U = 273.0	U = 155.0	U = 156.0	U = 16	U = 32.0
healthy<and<NOMGD	*p* = 0.04	*n.s.*	*n.s.*	*p* = 0.007	*p* = 0.01	*p* <0.001	*p*<0.001
Differences<between	U = 0.0	U = 10.0	U = 26.0	U = 56.0	U = 110.0	U = 30	U = 192.0
MGD<and<NOMGD	*p*<0.001	*p*<0.001	*p*<0.001	*p*<0.001	*p*<0.02	*p*<0.001	*n.s.*
Differences<between	U = 7.5	U = 135	U = 104.0	U = 21.0	U = 0.0	U = 120	U = 0.0
ATD<and<MGD	*p*<0.001	*n.s.*	*n.s.*	*p*<0.001	*p*<0.001	*n.s.*	*p*<0.001
Differences<between	U = 0.0	U = 76	U = 78.0	U = 0.0	U = 0.0	U = 84	U = 0.0
ATD<and<NOMGD	*p*<0.001	*p*<0.001	*p*<0.001	*p*<0.001	*p*<0.001	*p*<0.001	*p*<0.001
Differences<between	U = 181.0	U = 225	U = 357.0	U = 171	U = 216.0	U = 16	U = 364.5
healthy<and<both^+^	*p*<0.001	*p*<0.001	*p*<0.001	*p*<0.001	*p*<0.001	*p*<0.001	*p* = 0.002

* Average ± standard deviation.

† Median (mode) ± Average Absolute deviation. Mode was added if different from the median.

OSDI  =  Ocular Surface Disease ndex; FBUT  =  fluorescein break-up time; FSOS  =  fluorescein staining of the ocular surface;

SVST  =  standardized visual scale test; CoL  =  clearance/turnover of lipids in human tears; TDI =  tear dysfunction index; Obstructive MGD  =  (obvious) obstructive meibomian gland dysfunction; U =  *post-hoc* Mann-Whitney U tests with Bonferroni correction; K =  Kruskal–Wallis statistic; *n.s.*  =  not significant;

**Table 2:** Percentage Distributions of the lipid-based tracer at the 5^th^, 10^th^ and 15^th^ minute.

	*Homogeneous* type at the 5^th^ minute*				*Vortex* type at the 5^th^ minute*
	Excellent	Good	Fair	Poor	
Healthy	0%	9.5%	76.2%	0%	14.3%
ATD	0%	0%	0%	100%	0%
MGD	0%	0%	0%	100%	0%
NOMGD	0%	15.4%	46.1%	38.5%	0%

	*Homogeneous* type at the 10^th^ minute				*Vortex* type at the 10^th^ minute
	Excellent	Good	Fair	Poor	
Healthy	9.5%	28.6%	47.6%	0%	14.3%
ATD	0%	0%	10%	90%	0%
MGD	0%	0%	0%	100%	0%
NOMGD	0%	23.1%	61.5%	15.4%	0%

	*Homogeneous* type at the 15^th^ minute				*Vortex* type at the 15^th^ minute
	Excellent	Good	Fair	Poor	
Healthy	61.9%	38.1%	0%	0%	0%
ATD	0%	0%	20%	80%	0%
MGD	0%	0%	0%	100%	0%
NOMGD	0%	15.4%	69.2%	15.4%	0%

* Immediately after tracer instillation (within 30 seconds), the reflectivity of the lower tear meniscus was high/intense (grade 4 or poor, homogeneous pattern) in all cases (100%).

ATD  =  aqueous tear deficiency; MGD  =  obvious obstructive meibomian gland disease; NOMGD  =  non-obvious meibomian gland obstruction.

The differences in CoL among the four groups were found to be statistically significant (Kruskal–Wallis statistic, 65.8; *p*<0.001; Table 1). However, no significant differences in CoL were found between ATD and obstructive MGD (*post-hoc* Mann–Whitney *U* test with Bonferroni correction, 120.0; *p* = 0.07; Table 1).

### 2. TDI

The cutoff value, derived from the ROC curve, is the point with an *optimal relationship* between sensitivity and specificity. The best cutoff point (y) for TDI was calculated to be 1.35. By applying the y for TDI, the differences among the four groups were raised significantly (Kruskal–Wallis statistic, 57.30; *p*<0.001; Table 1). Interestingly, no significant differences in TDI were found between obstructive MGD and NOMGD (*post-hoc* Mann–Whitney *U* test with Bonferroni correction, 192.0; *p* = 0.92; Table 1) since both MGDs (obstructive MGD or NOMGD) are involved in determining a lipid alteration.

Gender did not influence the results (Kruskal–Wallis statistic, 0.009; *p* = 0.92).

### 3. Dynamic variations of the tear film and of the ocular surface

The vortex-pattern was observed only at the 5^th^ minute (14.3%) and 10^th^ minute (14.3%) in healthy subjects; in all other cases, the homogeneous pattern was detected (see Table 2). The results of correlation analyses are presented in Table 3.

**Table 3:** Correlation Coefficients and Statistical Significance (*p*) between Clearance of lipids in human tears, and Ocular Surface Disease Index, Fluorescein Break-up time, grade of Fluorescein Staining of the ocular surface, grade of Standardized Visual Scale Test, Schirmer I Test, and age.

All participants	Clearance of lipids (CoL) in human tears
OSDI	*r_s_ = 0.79*
	*p<0.001*
FBUT	*r_s_ = −0.68*
	*p<0.001*
Fluorescein Staining of the ocular surface (grade)	*r_s_ = 0.58*
	*p<0.001*
Standardized Visual Scale test (grade)	*r_s_ = 0.68*
	*p<0.001*
Schirmer I Test	*r_s_ = −0.5*
	*p<0.001*
Age	*r_s_ = −0.43*
	*n.s.*

Correlation coefficients (*r*_s_ ***= *** Spearman correlation coefficient). OSDI  =  Ocular Surface Disease Index; FBUT  =  fluorescein break-up time; SVST *** = *** standardized visual scale test; *n.s.* ***= *** not significant.

The differences over time of CoL and SVST are provided in Table 2 and 4. In all groups, the CoL has been slower than the aqueous flow, as assessed by SVST. Interestingly, a biphasic decay of the reflectivity elicited by the tracer was observed in some cases: after a dramatic initial dilution of tracer, probably due to the reflex tearing, there was a slower washout of lipids, probably due to the progressive reduction of tear volume. However, in case of healthy individuals, a highly significant clearance of tracer was restored after the 10^th^ minute.

**Table 4:** Statistical Differences for Clearance of lipids (CoL) in human tears and Standardized Visual Scale Test (SVST) within and between the four Groups: Healthy Persons, Aqueous Tear Deficiency (ATD), obvious obstructive Meibomian gland Dysfunction (MGD) and non-obvious obstructive Meibomian gland Dysfunction (NOMGD) Patients.

	Differences of CoL						
Intervals (minute)	Grade variations (percentage points)				Overall change (%)	Friedman test and Wilcoxon Signs Ranks test	Statistical Significance (*p*)
	Excellent*	Good	Fair	Poor			
**ALL patients** †							
**t = 0 and the 5^th^**	+3.7	+7.3	+34.1	−45.1	−14.9%	χ _F_ = 37.0	<0.001
**the 5^th^ and the 10^th^**	+2.4	+7.3	0	−9.8	−6.4%	χ _F_ = 16.0	<0.001
**the 10^th^ and the 15^th^**	+9.8	0	−7.3	−2.4	−6.8%	χ _F_ = 14.2	<0.001
**t = 0 and the 15^th^**	+15.9	+14.6	+26.8	−57.3	−25.9%	χ _F_ = 47.0	<0.001
**HEALTHY subjects** †							
**t = 0 and the 5^th^**	+14.3	+9.5	+76.2	−100	−34.5%	Z = −4.2	<0.001
**the 5^th^ and the 10^th^**	+9.5	+19.1	−28.6	0	−14.5%	Z = −2.2	= 0.02
**the 10^th^ and the 15^th^**	+38.1	+9.5	−47.6	0	−38.2%	Z = −3.8	<0.001
**t = 0 and the 15^th^**	+61.9	+38.1	0	−100	−65.4%	Z = −4.1	<0.001
**ATD patients** †							
**t = 0 and the 5^th^**	-	-	-	0	0%	Z = 0.0	n.s.
**the 5^th^ and the 10^th^**	-	-	+10	−10	−2.5%	Z = −1.4	n.s.
**the 10^th^ and the 15^th^**	-	-	+10	−10	−2.5%	Z = −1.4	n.s.
**t = 0 and the 15^th^**	-	-	+20	−20	−2.5%	Z = −2.0	<0.04
**MGD patients** †							
**t = 0 and the 5^th^**	-	-	-	0	0%	Z = 0.0	n.s.
**the 5^th^ and the 10^th^**	-	-	-	0	0%	Z = 0.0	n.s.
**the 10^th^ and the 15^th^**	-	-	-	0	0%	Z = 0.0	n.s.
**t = 0 and the 15^th^**	-	-	-	0	0%	Z = 0.0	n.s.
**NOMGD patients** †							
**t = 0 and the 5^th^**	-	+16	+44	−60	−19%	Z = −3.5	<0.001
**the 5^th^ and the 10^th^**	-	+16	+16	−44	−9.8%	Z = −2.8	= 0.005
**the 10^th^ and the 15^th^**	-	0	+8	0	−3.4%	Z = −0.6	n.s.
**t = 0 and the 15^th^**	-	+16	+68	−84	−25%	Z = −4.2	<0.001


	Differences of SVST			Differences between CoL-SVST	
Intervals (minute)	Overall change (%)	Friedman test and Wilcoxon Signs Ranks test	Statistical Significance (*p*)	Cochran's test and McNemar's test	
**ALL patients** †					
**t = 0 and the 15^th^**	−46.69%	χ _F_ = 76.0	<0.001	χ _C_ = 7.6	= 0.006
**HEALTHY subjects** †					
**t = 0 and the 15^th^**	−72.1%	Z = −4.1	<0.00	χ _M_ ††	n.s.
**ATD patients** †					
**t = 0 and the 15^th^**	−14.2%	Z = −3.3	= 0.001	χ _M_ ††	<0.001
**MGD patients** †					
**t = 0 and the 15^th^**	−36.1%	Z = −3.5	<0.001	χ _M_ ††	= 0.002
**NOMGD patients** †					
**t = 0 and the 15^th^**	−57.7%	Z = −4.4	<0.001	χ _M_ ††	n.s.

*  =  Excellent or Vortex-pattern;

† =  Immediately after tracer instillation (t = 0), the reflectivity of the lower tear meniscus was high/intense (grade 4 or poor, homogeneous pattern) in all cases (100%); -  =  Absent in the two scans performed at different times (subsequent serial scans or performed at t = 0 and the 15^th^).

†† =  Binomial distribution use; CoL  =  clearance of tear lipids; SVST  =  standardized visual scale test; *n.s.* =  not significant; Z =  Wilcoxon Signs Ranks test; χ _F_ =  Friedman test; χ _C_ =  Cochran's test; χ _M_ =  McNemar's test.

### 4. Diagnostic validity of the novel OCT technique

We have considered the values of CoL as *abnormal* when they were fair or poor, and *normal* when values were excellent or good, or when it was present a vortex pattern. The individual AUCs of CoL calculated by the ROC technique ranged from 0.66 to 0.98, suggesting reliable sensitivity and specificity of lipid-enhanced OCT imaging (Tables 5 and 6). Particularly, the AUC of CoL in the differential diagnosis between healthy subjects and patients with both abnormalities of the ocular surface (ATD + MGDs), was 0.98 ( p<0.001), asymptotic 95% confidence interval (0.0, 1.0). Again, the TDI showed a promising ability in differentiating ATD patients than patients with lipid deficiency due to MGDs, AUC = 1.00 ( p<0.001), asymptotic 95% confidence interval (0.0, 1.0).

**Table 5:** The diagnostic validity of the clearance of lipids (CoL) in human tears, considering the values abnormal when fair or poor, and normal when excellent or good (for the homogeneous type) or in case of the vortex pattern.

	Sensitivity	Specificity	Accuracy	PPV	NPV
**Healthy** †	**93.6%**	**100%**	**97.5%**	**100%**	**84%**
**ATD**	**48.7%**	**32.2%**	**50%**	**35%**	**100%**
**Obstructive MGD**	**100%**	**33.8%**	**43.9%**	**26.7%**	**100%**
**NOMGD**	**51.1%**	**84%**	**52.4%**	**84.6%**	**37.5%**
**Vortex pattern** † (at the 5^th^ and 10^th^ minute)	**14.2%**	**100%**	**96.3%**	**100%**	**77.2%**

† In this case, no abnormalities of the ocular surface (ATD, MGD/NOMGD)  =  positive diagnosis.

PPV  =  positive predictive value; NPV  =  negative predictive value; ATD  =  aqueous tear deficiency; obstructive MGD  =  (obvious) obstructive meibomian gland dysfunction; NOMGD  =  non-obvious obstructive meibomian gland dysfunction.

**Table 6:** The AUCs (areas under the curves) analyzed by receiver operating characteristics (ROC) curves.

Diagnosis (or Differential Diagnosis)	Area under the ROC curve (AUC)	Statistical Significance (*p*)	Asymptotic 95% Confidence Interval	
			Lower Bound	Upper Bound
***DD:.*** ** Healthy or abnormalities of the ocular surface** †	**0.98**	***P*** **<0.001**	**0.00**	**1.00**
**ATD**	**0.78**	***p***<**0.001**	**0.68**	**0.88**
**MGDs**	**0.66**	***p*** ** = 0.013**	**0.53**	**0.78**
***DD:.*** ** ATD or MGDs**	**0.67**	***p*** ** = 0.025**	**0.54**	**0.81**
**By applying the TDI (cutoff = 1.35) in the**	**1.00**	***p*** **<0.001**	**1.00**	**1.00**
***DD*** ** between ATD from MGDs**				

† In this case, no abnormalities of the ocular surface (ATD + MGDs)  =  positive diagnosis.

*DD* =  differential diagnosis. ATD  =  aqueous tear deficiency; obstructive MGD  =  (obvious) obstructive meibomian gland dysfunction; NOMGD  =  non-obvious obstructive meibomian gland dysfunction; MGDs  =  obstructive MGD or NOMGD; ROC curves  =  (receiver operating characteristics) curves.

The sensitivity of the CoL in diagnosing healthy (only in this case, healthy subject  =  positive diagnosis), obstructive MGD, NOMGD, ATD, was 93.6%, 100%, 51.1%, 48.7%, respectively. The specificity was 100%, 33.8%, 84%, 36.2%, respectively. The diagnostic accuracy was 97.5%, 43.9%, 52.4%, 50%, respectively. Moreover, the positive predictive value (PPV) was 100%, 26.7%, 84.6%, 35%, respectively, and the negative predictive value (NPV) was 84%, 100%, 37.5%, 100%, respectively.

By applying the TDI (cut-off point at 1.35), the sensitivity of lipid-enhanced OCT imaging in diagnosing ATD or MGDs was raised to 100%.

The sensitivity, specificity, accuracy, PPV and NPV of the vortex-pattern (at the 5^th^ and 10^th^ minute) in diagnosing healthy patients (in this case, healthy  =  positive diagnosis) was 14.2%, 100%, 96.3%, 100%, 77.2%, respectively).

## Discussion

The lipids found in human tears are numerous and exert several functions. [29] The meibomian gland secretes a complex mixture comprising families of non-polar lipids, polar lipids, and proteins. Although over one hundred major compounds have been identified, Tiffany estimated that there could be thousands yet undiscovered. [30], [31] In the outer layer of the tear film – the lipid layer – there is a polar phase, which has surfactant properties, and a non-polar phase, which retards water vapor transmission. Although considerable importance has been given to the study of lipid component of tears in the understanding of dry eye, the degree of washing out of the lipids in ATD, obstructive MGD, NOMGD patients, remains unknown.

In the present study, we have used an oil-in-water emulsion as an OCT tracer for analyzing the dynamic distribution of lipids in human tears. The degree of washing out of tracer, graded according to the OCT reflectivity, was considered an index of turnover of tear lipids. Since the lower tear meniscus shows a reflectivity absent (or nearly undetectable) at baseline, the signal intensity detected by OCT, after instillation of tracer, is directly dependent on its amount on the ocular surface. Thus, our method has allowed us to study in vivo the behavior of lipids and their role in the complex dynamics of fluids of the lacrimal system.

Although lipids are distributed in the outer layer of the precorneal tear film, it has been possible to document their distribution in the tear meniscus, which appears as a vortex pattern. For its dynamic characteristics, the tear meniscus may function both as reserve and as scavenger for lipids. In fact, the vortex pattern has allowed highlighting the presence of centrifugal forces that push the tears outside, toward the lid skin. It is, therefore, possible to hypothesize that the mechanism of excretion of tear lipids toward the lid skin occurs following these centrifugal forces (and not only for an overflow due to a large tear volume). [1], [12] This condition has been well evident in the case of high fluid dynamics, which has occurred only in healthy patients. Since the turnover of lipids in tears has been slower than the clearance of aqueous tear (SVST) in all groups, and because of water is non-reflective whereas lipids are hyper-reflective, the hypo-reflective substances at the center of the vortex, rinsed faster, are clearly those of the aqueous layer. In case of abnormality of the ocular surface, we observed a less dynamic distribution of lipids in the meniscus and a slower washout of lipids (CoL). For all these reasons, we believe that there are different routes of lipid excretion from tears. Probably, the biochemical interactions between lipids and proteins in the aqueous layer (e.g., lipocalin) may have a role in the mechanism of excretion, [13], [14], [32]–[34] and transfer some lipids in the aqueous flow, at the center of the vortex.

Although previous studies have documented the importance of tear lipids, our work revealed a prolonged retention time of tear lipids in abnormal eyes. Since tear lipids can easily coalesce to form droplets because of their thermodynamic instability, it follows that the slower the CoL, the greater the alteration of their stability and their functions.

Moreover, the results of our research indicate the existence of a correlation between the turnover of aqueous tear, as assessed by SVST, and the CoL. In fact, patients with high aqueous flow tend to have a faster washout of lipids and, conversely, patients with slow aqueous tear turnover (e.g., ATD) have more difficulty in excretion of lipids from tears. Again, a slower CoL has also been found in individuals with MGDs. Probably, alterations of lipids induce an increase of evaporation such as to reduce the dynamics of fluids of the ocular surface. In fact, several abnormalities of tear lipids in MGD patients have been found. For instance, variations in the conformation of lipids due to the changes of amount of lipid saturation governing lipid-lipid strength, or an increased viscosity due to an increase in protein and others compositional differences of *meibum* (also known as “meibomian gland secretions”). It is interesting to note that patients with NOMGD, despite the minimal signs of their dysfunction, already show a slower CoL.

A significant correlation with others traditional tests and a promising diagnostic validity of turnover of tear lipids (CoL) has been revealed in our work. The CoL can be used in the differential diagnosis between healthy and ATD/MGDs individuals, and may be useful in providing a permanent record, which permits masking of scoring and therefore provides greater objectivity. Such records can be handled at a reading center, to provide improved standardization in clinical trials. By applying the TDI, it is also possible to clarify the impact of a lipids alteration or an aqueous deficiency, and even differentiate the two forms. Therefore, based on the clinical results and statistical significance of specificity, sensibility, PPV, NPV and accuracy, we believe that CoL can be considered a useful test for the diagnosis and follow-up of dry eye patients.

Although the fluorophotometric measurement using fluorescein sodium as a tracer has been the gold standard to quantify tear flow, [13], [14], [35] since the report of Mishima et al, [11] the analysis of lipids turnover by using OCT (compared to the fluorometer) has several advantages: it allows a morphological evaluation of the dynamic distribution of lipids; it does not require fluorescein sodium, which destabilizes the tear film, can penetrate through the cornea, and may create staining and pseudo-staining of the ocular surface; it provides final results that are not influenced by the autofluorescence of the cornea; lipid-enhanced OCT imaging is easier to perform because it is more practical to obtain a drop (∼35 µl) of tracer that 5 µl of fluorescein sodium with a micropipette; it provides an objective and permanent record, important for the patient follow-up and the analysis among multiple examiners.

The current study shows that the lipids contribute largely to the increase of OCT reflectivity of the tear meniscus, which is otherwise non-reflective (or nearly undetectable). To the best of our knowledge, our study is the first that describes the use of a tracer to enhance the reflectivity of OCT images and to obtain an increase in the contrast and visibility of scanned structures. This finding could be of clinical relevance in the interpretation of OCT images and may be used for the construction of new contrast media for OCT technologies.

There are potential limitations when interpreting the results of the present study. The turnover of the tracer used in our work can be considered a surrogate measure of the tear lipids washout. In fact, in the future, when all the tear lipids will be known, a tracer most similar to the lipid layer (with all compounds, in physiological proportions) could be built and used to obtain a new *non-specific* index of CoL. On the other hand, further studies could evaluate the different routes of excretion using different *specific* tracers for *each lipid*. Moreover, a more comprehensive database of normal individuals must be created to analyze possible age- and sex-dependent variations.

## Conclusion

In conclusion, lipid-enhanced OCT imaging is a promising method to detect changes of the tear film and of the ocular surface. Assessment of the dynamic distribution of the tear lipids may improve the clinician's ability in achieving significant information regarding the complex fluid dynamics of human tears and in correctly classifying the dry eye syndrome. Moreover, this technique should facilitate the widespread use of the clearance of tear lipids for diagnosis and could be useful for the identification and follow-up of patients with specific abnormalities of the ocular surface, in particular with NOMGD.
